# A Novel Mechanism of Host-Pathogen Interaction through sRNA in Bacterial Outer Membrane Vesicles

**DOI:** 10.1371/journal.ppat.1005672

**Published:** 2016-06-13

**Authors:** Katja Koeppen, Thomas H. Hampton, Michael Jarek, Maren Scharfe, Scott A. Gerber, Daniel W. Mielcarz, Elora G. Demers, Emily L. Dolben, John H. Hammond, Deborah A. Hogan, Bruce A. Stanton

**Affiliations:** 1 Department of Microbiology and Immunology, The Geisel School of Medicine at Dartmouth, Hanover, New Hampshire, United States of America; 2 Genome Analytics Helmholtz Centre for Infection Research, Braunschweig, Lower Saxony, Germany; 3 Departments of Genetics and Biochemistry, The Geisel School of Medicine at Dartmouth, Hanover, New Hampshire, United States of America; 4 Norris Cotton Cancer Center, Lebanon, New Hampshire, United States of America; The University of Texas at Austin, UNITED STATES

## Abstract

Bacterial outer membrane vesicle (OMV)-mediated delivery of proteins to host cells is an important mechanism of host-pathogen communication. Emerging evidence suggests that OMVs contain differentially packaged short RNAs (sRNAs) with the potential to target host mRNA function and/or stability. In this study, we used RNA-Seq to characterize differentially packaged sRNAs in *Pseudomonas aeruginosa* OMVs, and to show transfer of OMV sRNAs to human airway cells. We selected one sRNA for further study based on its stable secondary structure and predicted mRNA targets. Our candidate sRNA (sRNA52320), a fragment of a *P*. *aeruginosa* methionine tRNA, was abundant in OMVs and reduced LPS-induced as well as OMV-induced IL-8 secretion by cultured primary human airway epithelial cells. We also showed that sRNA52320 attenuated OMV-induced KC cytokine secretion and neutrophil infiltration in mouse lung. Collectively, these findings are consistent with the hypothesis that sRNA52320 in OMVs is a novel mechanism of host-pathogen interaction whereby *P*. *aeruginosa* reduces the host immune response.

## Author Summary

*Pseudomonas aeruginosa* is a gram-negative, opportunistic pathogen that accounts for about 10% of all hospital-acquired infections in the US and primarily infects immunocompromised hosts, including patients with chronic obstructive pulmonary disease and cystic fibrosis. Gram-negative bacteria like *P*. *aeruginosa* produce outer membrane vesicles (OMVs), which constitute an important mechanism for host colonization. In this study we demonstrate a novel mechanism of pathogen-host interaction that attenuates the innate immune response in human airway epithelial cells and in mouse lung through a regulatory sRNA contained inside OMVs secreted by *P*. *aeruginosa*.

## Introduction

*Pseudomonas aeruginosa* is a gram-negative, opportunistic pathogen that primarily infects immunocompromised hosts including cancer and AIDS patients, burn victims, patients on ventilators and individuals with chronic obstructive pulmonary disease and cystic fibrosis. According to the Centers for Disease Control and Prevention (CDC) 51,000 people each year contract a hospital-acquired *P*. *aeruginosa* infection in the U.S., accounting for about 10% of all nosocomial infections [1]. An estimated 6,700 of these infections are caused by multidrug-resistant *P*. *aeruginosa* strains, which have a very high mortality rate and have therefore been rated as a serious threat by the CDC (CDC, 2013).

Like other gram-negative bacteria, *P*. *aeruginosa* produces outer membrane vesicles (OMVs), which constitute an important mechanism of interaction with hosts and competing bacterial strains in their natural environment [2,3]. OMVs are 50–250 nm spheroid particles derived from the outer membrane that are constitutively secreted and consist of lipids, proteins and lipopolysaccharide (LPS) [3]. OMVs of many gram-negative bacteria including *P*. *aeruginosa* also contain DNA [4,5]. OMVs are involved in quorum sensing and enable bacteria to establish a colonization niche by selectively killing or promoting the growth of other bacteria, and they transmit virulence factors and toxins to host cells, thereby modulating the host immune response [2,6–10]. Distinct *P*. *aeruginosa* virulence factors like alkaline phosphatase, phospholipase Cs, β-lactamase and Cif (CFTR Inhibitory Factor) are differentially packaged and enriched in OMVs [11,12]. OMVs diffuse across mucus and fuse with airway epithelial cells releasing their cargo into host cells [11,13].

OMVs elicit a pro-inflammatory host immune response through pathogen-associated molecular patterns (PAMPs) [14,15]. PAMPs, including LPS, peptidoglycan, flagellin, porins and lipoproteins interact with Toll-like receptors (TLR) in host cells, which signal through mitogen-activated protein kinases (MAPK) leading to increased secretion of pro-inflammatory cytokines, notably IL-8 in the case of human airway epithelial cells [14,16]. Cytokine secretion rapidly attracts neutrophils and macrophages to the site of infection [17], leading to bacterial clearance in most cases. To establish a chronic infection, *P*. *aeruginosa* deploys several strategies that mediate host immune system evasion in the various stages of the colonization process. These strategies include up-regulation of the production of polysaccharide and alginate, down-regulation of virulence factor expression, reduced phagocytic uptake of *P*. *aeruginosa* by immune cells and elimination of flagellar motility and conversion to a mucoid, sessile lifestyle [18]. In addition, OMV-mediated mechanisms also counteract the host immune response to *P*. *aeruginosa*. The OMV virulence factor Cif (PA2934), for example, dampens the airway innate immune response by promoting lysosomal degradation of CFTR, which reduces chloride secretion and thereby the airway hydration that is essential for mucocilliary clearance of pathogens [19]. Furthermore, Cif increases degradation of the transporter associated with antigen processing 1 (TAP1) and decreases major histocompatibility complex (MHC) class 1 antigen presentation in airway epithelial cells, thus attenuating the adaptive immune response to viral infection [20]. OMVs may also act as decoys that absorb anti-microbial compounds produced by the host [8], and allow bacteria to evade immune detection during colonization, as has been shown for *Neisseria gonorrhoeae* [21]. *Porphyromonas gingivalis* packages gingipains into OMVs, which degrade cytokines, hence down-regulating the host immune response [22].

It is well established that intracellular small RNAs (sRNAs) have regulatory functions in *P*. *aeruginosa* [23–25] and other bacterial species [26]. sRNAs regulate cell envelope structure, metabolism, bacterial communication, quorum sensing, biofilm formation and virulence [23,25,27]. The regulatory mechanism involves either binding to target bacterial mRNAs, thereby affecting translation, or direct interaction with protein targets [28]. A particular group of regulatory sRNAs, transfer RNA (tRNA) fragments, has gained recognition as important biological regulators in many prokaryotic and eukaryotic species [29,30]. In contrast to classic bacterial sRNAs, which are very target-specific, tRNA fragments are thought to repress translation in a manner similar to microRNAs, which often regulate multiple mRNA targets [31,32].

Studies on bacterial regulatory sRNAs have focused on describing their endogenous effects, while their inter-species effects have remained largely unknown. Three recent reports describe the RNA content of OMVs secreted by *E*. *coli*, *P*. *gingivalis* and *V*. *cholerae* [33–35], but no biological effects on host cells were reported. Accordingly, the primary aim of this study was to test the hypothesis that *P*. *aeruginosa* OMVs contain sRNAs that impact human cells in biologically important ways. Here, we provide a first characterization of *P*. *aeruginosa* sRNAs in OMVs and demonstrate that a specific bacterial sRNA (sRNA52320) is transferred from OMVs to host cells, where it attenuates OMV-stimulated IL-8 secretion by human airway epithelial cells, and KC cytokine secretion and neutrophil recruitment in the lungs of a mouse model.

## Results

### OMVs contain differentially packaged sRNAs

**Fig 1:**
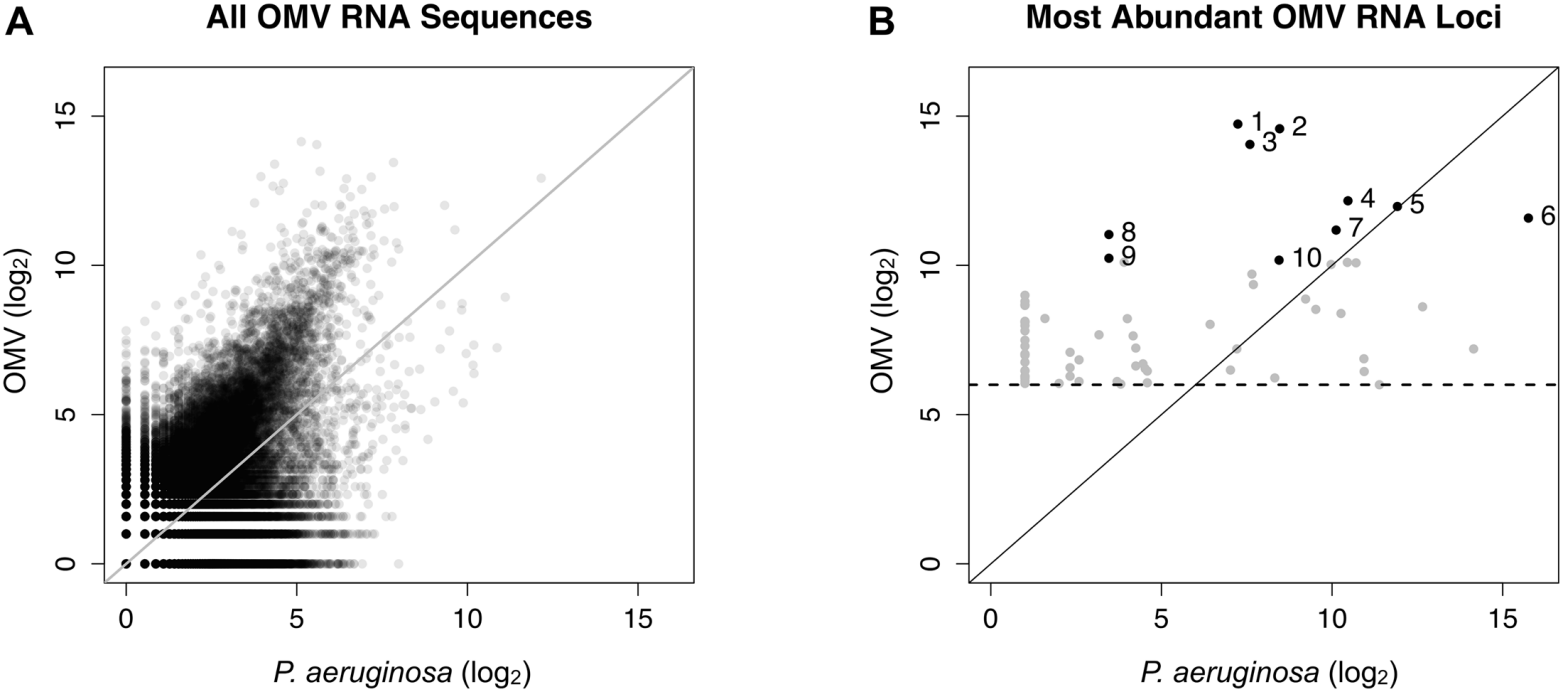
OMVs contain differentially packaged sRNAs. (A) Relative abundance of 481,480 unique OMV sRNA sequence reads versus *P*. *aeruginosa* whole cell sRNA sequence reads. Sequences to the left of the solid diagonal line were more abundant in OMVs than in whole cells and sequences to the right were less abundant in OMVs. The median sequence length of the sRNAs was 24 nucleotides, with a minimum of 15 and a maximum of 45 nucleotides. (B) Relative abundance of sRNAs associated with a locus in OMVs versus *P*. *aeruginosa*. The 1733 most abundant OMV sRNA sequences (with a minimum of 64 counts, indicated by the horizontal dashed line) mapped to 68 PA14 loci. Loci to the left of the solid diagonal line were more abundant in OMVs than in *P*. *aeruginosa*, while those to the right of the line were less abundant in OMVs than in *P*. *aeruginosa*. On average, loci that were abundant in OMVs were 9-fold enriched in OMVs compared to *P*. *aeruginosa* (mean log_2_ Ratio = 3.17, 95% CI = 2.28–4.06, p = 1.1*10^−9^). Significance was determined using a one-sample t-test. The 10 most abundant OMV loci (solid black circles) are numbered and annotated in more detail in Table 1.

RNA-Seq analysis of *P*. *aeruginosa* and OMVs was conducted to determine if sRNAs are packaged in OMVs. We identified 481,480 unique sRNA sequences in OMVs purified from the supernatants of three planktonic cultures of *P*. *aeruginosa* strain PA14 (Fig 1A). The median sequence length of the sRNAs was 24 nucleotides, with a minimum of 15 and a maximum of 45 nucleotides. Replicate samples from three separate cultures were highly reproducible and correlated well with each other (r = 0.97).

The 1733 most abundant sRNA sequences in OMVs (i.e., at least 64 sequence reads) mapped to 68 loci in the *P*. *aeruginosa* genome, many of which represented by similar reads of different lengths. Fifty-two loci were at least twofold enriched in OMVs compared to *P*. *aeruginosa* whole cells (Fig 1B), while eight loci were at least twofold less abundant in OMVs compared to *P*. *aeruginosa*, and eight loci were about equally abundant. Relative abundance of loci read counts in OMVs compared to *P*. *aeruginosa* was very consistent across replicate samples, with eight of the ten most abundant loci being enriched in OMVs (Fig 1B and Table 1).

**Table 1:** Several sRNAs in OMVs are predicted to target human immune mRNAs.

#^a^	PA14 Locus	Function	OMV counts^b^	log_2_ Ratio^c^	Stable secondary structure?	Predicted human immune target?
1	*50830*	hypothetical protein	27202	**7.49**	Y	Y
2	*15350*	integrase	24424	**6.11**	N	Y
3	*41210*	DNA-binding protein hupB	16968	**6.46**	N	N
4	*28740*	tRNA-Pro	4583	**1.70**	N	N
5	*59370*	pathogenicity island	4015	0.06	Y	Y
6	*51240*	purine biosynthesis enzyme purC	3067	-4.17	Y	N
7	*52320*	tRNA-Met	2321	**1.06**	Y	Y
8	*44960*	hypothetical protein	2094	**7.54**	N	Y
9	*41340*	tRNA-Arg	1207	**6.74**	N	Y
10	*62790*	tRNA-Met	1155	**1.73**	Y	Y

^a^The numbers in column 1 correspond to the labels in Fig 1B.

^b^OMV counts show the total number of reads for each locus in OMV-derived sRNAs.

^c^The log_2_ ratio is the number of total reads in OMVs divided by the number of total reads in *P*. *aeruginosa* whole cells for each locus. Loci with more counts in OMVs than *P*. *aeruginosa* are highlighted in bold.

### Several sRNAs in OMVs are predicted to target human immune mRNAs

We used bioinformatic approaches to determine if the ten most abundant sRNAs in OMVs were likely to form stable secondary structures, and to identify potential interactions between these sRNAs and human mRNAs (Table 1). The complementary sequences of the ten most abundant OMV sRNAs were aligned with NCBI human reference mRNA sequences using the algorithm BLASTN 2.2.31 [36] to identify perfect matches with human mRNAs. Matches with an E value < 12 and a connection to the innate immune response were considered potential host immune targets of the sRNAs (Table 1). We chose sRNA52320 (#7) as a candidate for further analysis because of its stable secondary structure, predicted targets, and sufficient length for specific detection with PCR primers. sRNA52320 is a tRNA fragment of the first 24 nucleotides of a tRNA coding for methionine (tRNA-Met). *P*. *aeruginosa* has three additional loci coding for tRNA-Met with identical anticodon loops, but distinct sequences in the 5’ and 3’ regions. S1 Fig presents RNA-Seq read alignments to the *PA14_52320* locus for sRNAs isolated from OMVs (S1A) and from *P*. *aeruginosa* (S1B).

### sRNA52320 is inside of OMVs and protected from RNase digestion

**Fig 2:**
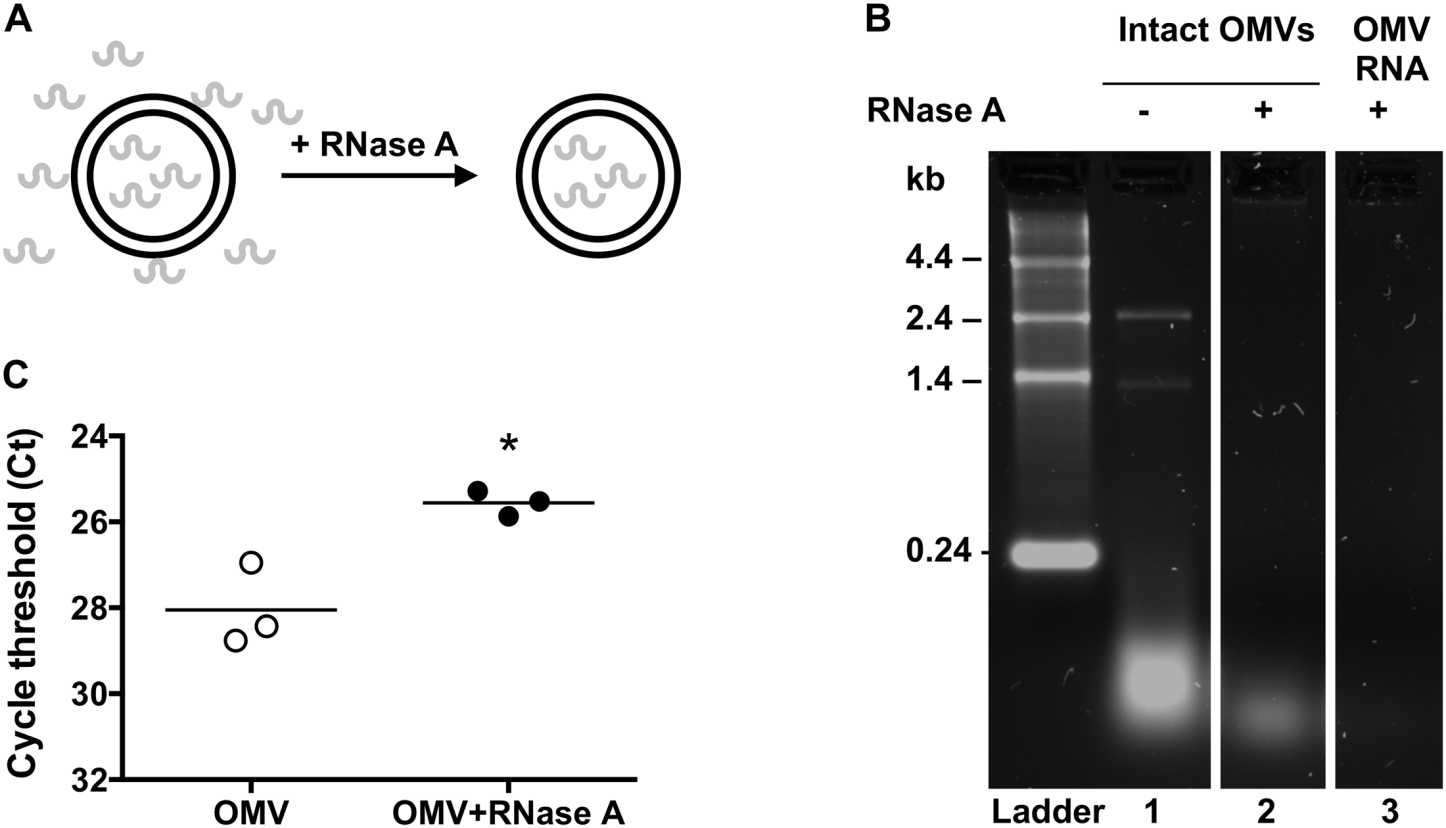
sRNA52320 is inside of OMVs and protected from RNase digestion. (A) RNase A digests free RNA including RNA associated with the outside of OMVs, while RNA inside of intact OMVs is protected from degradation. (B) Agarose gel showing profiles of OMV-associated RNAs from untreated control OMVs (lane 1), RNase A treated OMVs (lane 2) and OMV RNA extracted from QIAzol lysed OMVs after digestion with RNase A (lane 3). RNA was visualized by staining with SYBR Safe. Samples were run on the same gel and were re-arranged for presentation. (C) qPCR for sRNA52320 using RNA isolated from control OMVs or RNase A-treated OMVs. RNase A treatment prior to RNA-Isolation (filled circles) increased the relative abundance of sRNA52320 compared to untreated OMVs (open circles). The difference in mean cycle threshold (Ct) of -2.5 ± 0.6 was statistically significant (95% CI = -4.1 to -0.9, N = 3, p = 0.013 indicated by an asterisk).

To determine if sRNAs were inside OMVs, and not adherent to the outside of the OMV, studies were conducted with RNase A, a membrane-impermeable enzyme that will only degrade sRNAs adherent to the outside of OMVs (Fig 2A). In the absence of RNase A, RNA was associated with OMVs in various lengths, including 23S and 16S rRNA as well as many smaller RNAs 15–50 nt long (Fig 2B, lane 1). The RNA recovered from OMVs after RNase A digestion consisted predominantly of small RNAs around 15–50 nt (Fig 2B lane 2). As a control, RNase A completely digested RNA extracted from lysed OMVs (Fig 2B, lane 3). To determine if sRNA52320 was packaged inside OMVs, qPCR with primers specific for sRNA52320 was performed with RNA isolated from RNase-treated and untreated OMVs. RNase digestion increased the relative abundance of sRNA52320 in OMVs treated with RNase compared to OMVs exposed to vehicle (Fig 2C), confirming that sRNA52320 was inside the OMVs.

### sRNAs are transferred from OMVs to host cells

**Fig 3:**
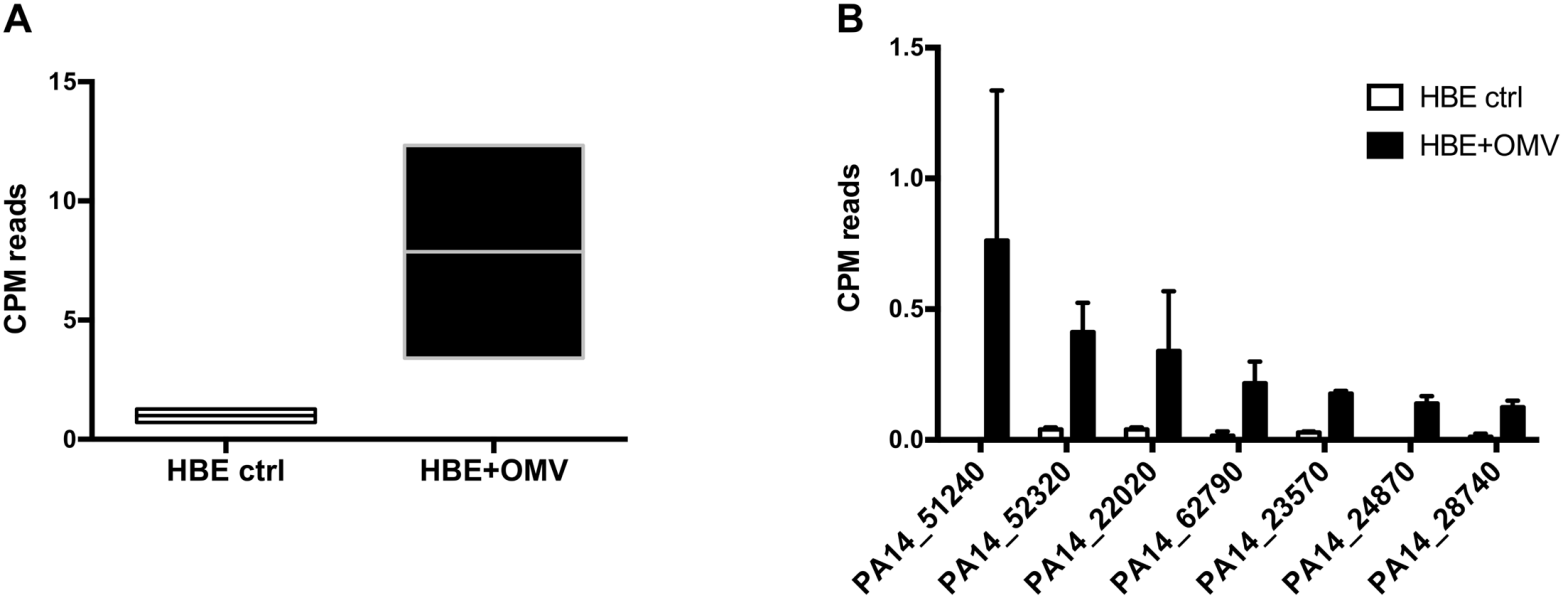
sRNAs are transferred from OMVs to host cells. (A) Counts per million (CPM) reads that uniquely aligned to the PA14 reference sequence in unexposed HBE cells (left, HBE ctrl) and HBE cells that had been exposed to OMVs and washed vigorously after exposure (right, HBE+OMV). (B) Counts per million (CPM) reads for the seven PA14 loci most abundantly detected in OMV-exposed HBE cells.

To determine if OMVs deliver their sRNA cargo to host cells, we performed RNA-Seq on primary human bronchial epithelial (HBE) cells that had been exposed to OMVs and on HBE cells that had not been exposed to OMVs. In samples from OMV-exposed HBE cells a detectable number of reads aligned to the PA14 reference genome, while RNA from unexposed HBE cells did not yield a signal above background levels (Fig 3A). sRNA52320 was one of the most abundant sRNAs in exposed host cells (Fig 3B). In addition, six other sRNAs were reliably detected in HBE cells exposed to OMVs.

### sRNA52320 targets kinases in the LPS-stimulated MAPK signaling pathway and is predicted to attenuate the innate immune response

**Fig 4:**
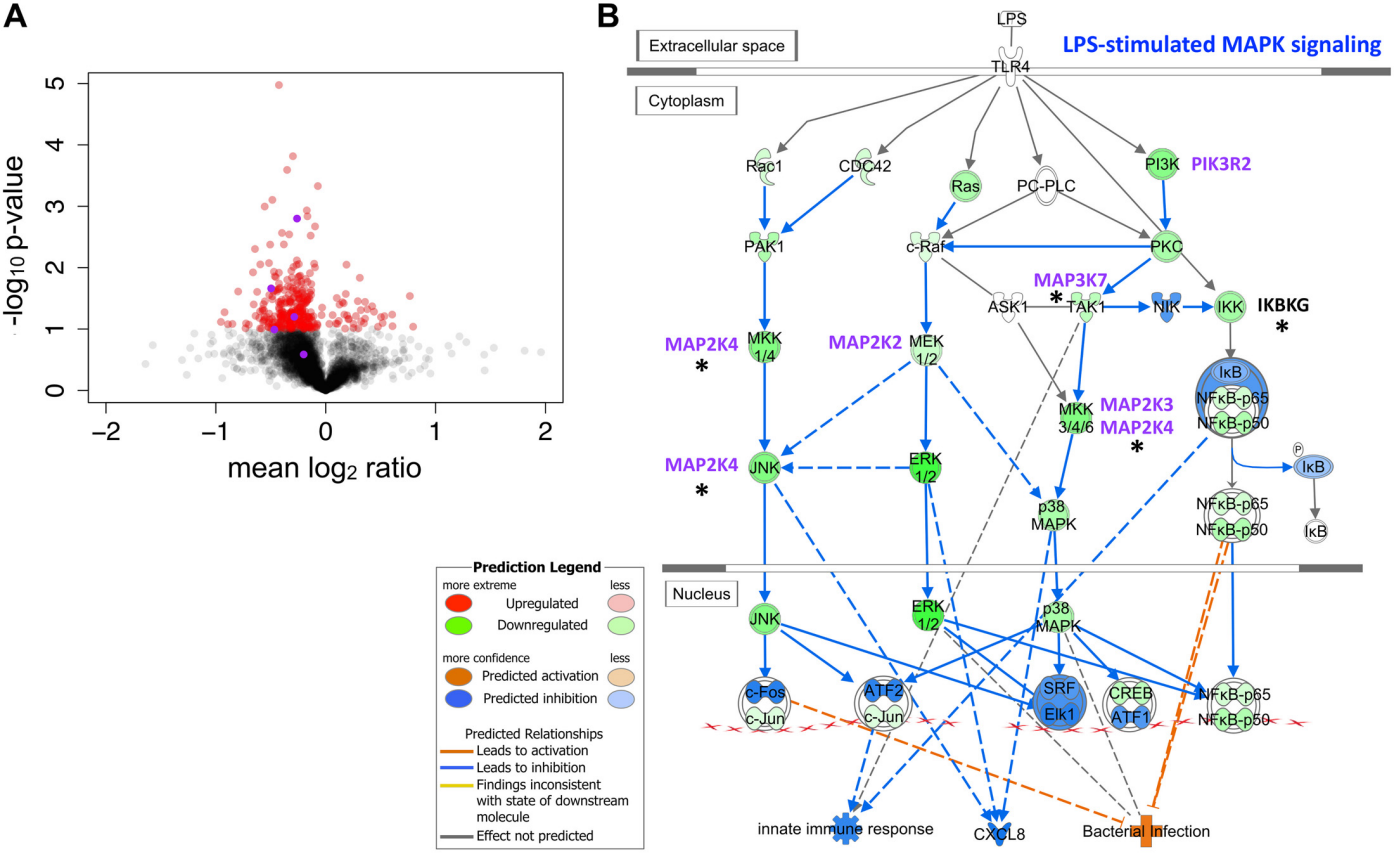
sRNA52320 targets kinases in the LPS-stimulated MAPK signaling pathway and is predicted to attenuate the innate immune response. (A) Volcano plot of -log_10_ p-values and mean log_2_ ratios for proteins from LPS-stimulated HBE cells transfected with sRNA52320 compared to LPS-stimulated HBE cells transfected with negative control RNA (siNC). A total of 3902 proteins were identified in all three HBE cell donor samples. Proteins that were differentially expressed (320 total), as determined by a one-sample t-test, are shown as red circles (darker shade indicates higher abundance). sRNA52320 decreased the abundance of the majority of the 320 proteins. (B) The 320 proteins whose abundance was altered by sRNA52320 were analyzed with Ingenuity Pathway Analysis (IPA). The depicted IPA canonical pathway “LPS-stimulated MAPK signaling” was one of the top 10 canonical pathways identified (p = 0.005, activation z-score = -2.236). The green circles identify proteins whose abundance was reduced by sRNA52320 compared to siNC. Five kinases in the LPS-stimulated MAPK signaling pathway were predicted to be direct targets of sRNA52320, including MAP2K2, MAP2K3, MAP2K4, MAP3K7, and PIK3R2 (indicted by purple text). *denotes significantly decreased protein abundance by sRNA52320 (p < 0.05). Blue shading indicates predicted inhibition and orange shading stands for predicted activation.

We speculated that bacterial sRNAs might function like eukaryotic microRNAs, which repress translation by imperfectly binding to many mRNA targets. To test this hypothesis, a miRanda microRNA target scan [37] was conducted and revealed that sRNA52320 is predicted to target mRNAs encoding multiple kinases in the LPS-simulated MAPK signaling pathway, including MAP2K2, MAP2K3, MAP2K4, MAP3K7 and PIK3R2. To identify proteins whose abundances were altered upon exposure to sRNA52320, a proteomic analysis was conducted on LPS-stimulated primary HBE cells transfected with synthesized sRNA52320 or a negative control RNA (siNC). Efficient transfection with sRNA52320 was verified for all samples by PCR as shown in S2A Fig Proteomic experiments with three biological replicates yielded 3902 quantifiable proteins. To select candidate proteins whose abundance were modified by sRNA52320, we chose the top 320 differentially expressed proteins by p-value. Differential expression ranged from 52% to 173% of control, with a majority of proteins down-regulated in the presence of sRNA52320 (Fig 4A). This broadly down-regulated protein expression profile is consistent with the hypothesis that sRNA52320 directly and/or indirectly represses many targets.

Using Ingenuity Pathway Analysis (IPA) to analyze the list of differentially expressed proteins, we identified ten pathways regulated by sRNA52320, including: Integrin Signaling, Rac Signaling, Signaling by Rho Family GTPases, Agrin Interactions at Neuromuscular Junction, Paxillin Signaling, Cdc42 Signaling, CXCR4 Signaling, GNRH Signaling, LPS-stimulated MAPK Signaling and HGF Signaling. Eight of these pathways are directly connected to the host immune response to pathogens and/or epithelial barrier function. As illustrated in Fig 4B, sRNA52320 down-regulated all detectable proteins in the LPS-stimulated MAPK signaling pathway, which IPA predicts to result in decreased IL-8 (CXCL8) levels, a reduced innate immune response, and consequently increased bacterial infection. sRNA52320 was predicted to down-regulate five kinases in the LPS-stimulated MAPK pathway in the bioinformatics analysis described above (highlighted in purple in Fig 4), and two of these kinases (MAP3K7 and MAP2K4) were significantly reduced by sRNA52320 (Fig 4B).

### Transfection with sRNA52320 reduces LPS-stimulation of IL-8 mRNA abundance and IL-8 cytokine secretion in HBE cells

**Fig 5:**
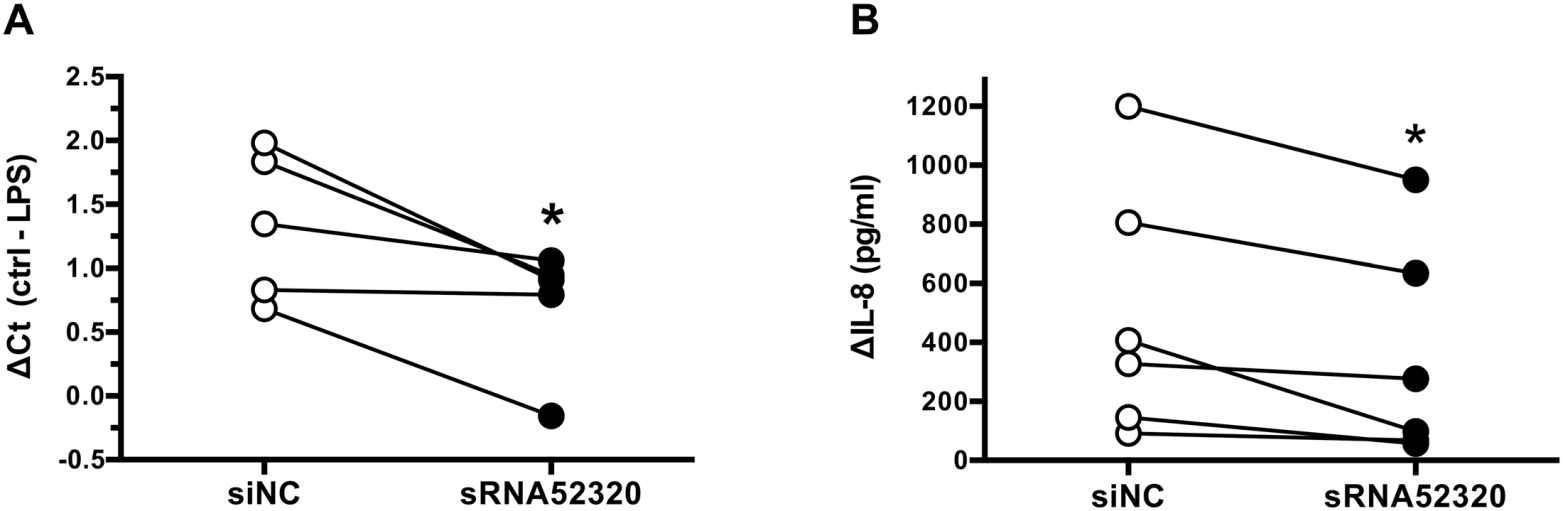
Transfection with sRNA52320 reduces LPS-stimulation of IL-8 mRNA abundance and IL-8 cytokine secretion in HBE cells. (A) HBE cells transfected with sRNA52320 (filled circles) had a lower induction of IL-8 mRNA (ΔCt = control Ct minus LPS-stimulated Ct) in response to LPS than control cells transfected with siNC (open circles). There was a statistically significant difference in the ΔCt means of -0.6 ± 0.2 (95% CI = -1.2 to -0.1, N = 5, p = 0.034 indicated by an asterisk). (B) sRNA52320 reduced IL-8 secretion by HBE cells in response to LPS. The difference in mean IL-8 secretion between cells transfected with siNC (open circles) and sRNA52320 (filled circles) of -149 ± 47 pg/ml was statistically significant (95% CI = -269 to -29, N = 6, p = 0.02 indicated by an asterisk).

Ingenuity pathway analysis of the data suggest that sRNA52320 will reduce IL-8 (CXCL8) levels in HBE cells (Fig 4B). To test the hypothesis that sRNA52320 reduces LPS-stimulated induction of IL-8 mRNA and IL-8 cytokine secretion, experiments were conducted on primary HBE cells transfected with sRNA52320 or siNC. As described above, efficient transfection with sRNA52320 was verified for all samples by PCR as shown in S2A Fig RT-PCR analysis of IL-8 revealed that sRNA52320 reduced the LPS-mediated induction of IL-8 mRNA compared to siNC (Fig 5A). Likewise, sRNA52320 reduced LPS-induced IL-8 protein secretion compared to siNC (Fig 5B).

To assess whether the mechanism of action of sRNA52320 requires sRNA52320 to be taken up into the host cell (rather than acting upon RNA-sensitive Toll-like receptors on the outside of the cell) [38], host cells were exposed to sRNA52320 in the presence and absence of transfection reagent. sRNA52320 could be detected in lysed HBE cells only when cells were exposed to sRNA52320 and the transfection reagent (S2A Fig). Importantly, sRNA52320 reduced LPS-stimulated IL-8 secretion only when it was transfected into HBE cells, but not when it was present exclusively outside of host cells (S2B Fig). These results support the hypothesis that sRNA52320 suppresses LPS-induced IL-8 secretion by interfering with translation of host mRNAs inside HBE cells rather than acting upon RNA-binding cell membrane receptors, such as TLR7/8.

### sRNA52320 reduces OMV-induced IL-8 secretion by HBE cells

To provide additional support for the observation that sRNA52320 delivered into host cells by OMVs reduces OMV-stimulated IL-8 secretion, we generated a *P*. *aeruginosa* deletion mutant for sRNA52320 as well as a re-complemented mutant that stably expressed sRNA52320 from a plasmid with an arabinose-inducible promoter (hereafter called ΔsRNA+sRNA). Deletion mutants were viable despite deletion of the tRNA-Met encoded by the *PA14_52320* locus due to the presence of three other redundant loci encoding tRNA-Met with the same anticodon. To allow for a direct isogenic comparison and account for potential effects of the vector or selective antibiotic, OMVs isolated from the re-complemented strain ΔsRNA+sRNA were compared to OMVs isolated from the deletion mutant transformed with an empty vector (hereafter called ΔsRNA+vector). Deletion and re-complementation of sRNA52320 in *P*. *aeruginosa* were verified by PCR (S3A Fig). sRNA52320 levels in re-complemented ΔsRNA+sRNA OMVs were similar to the amount of sRNA52320 naturally occurring in wt OMVs (S3B Fig). When comparing ΔsRNA+vector and ΔsRNA+sRNA OMVs there was no significant difference in the amount of LPS (S3C Fig) or protein content (S3D Fig).

**Fig 6:**
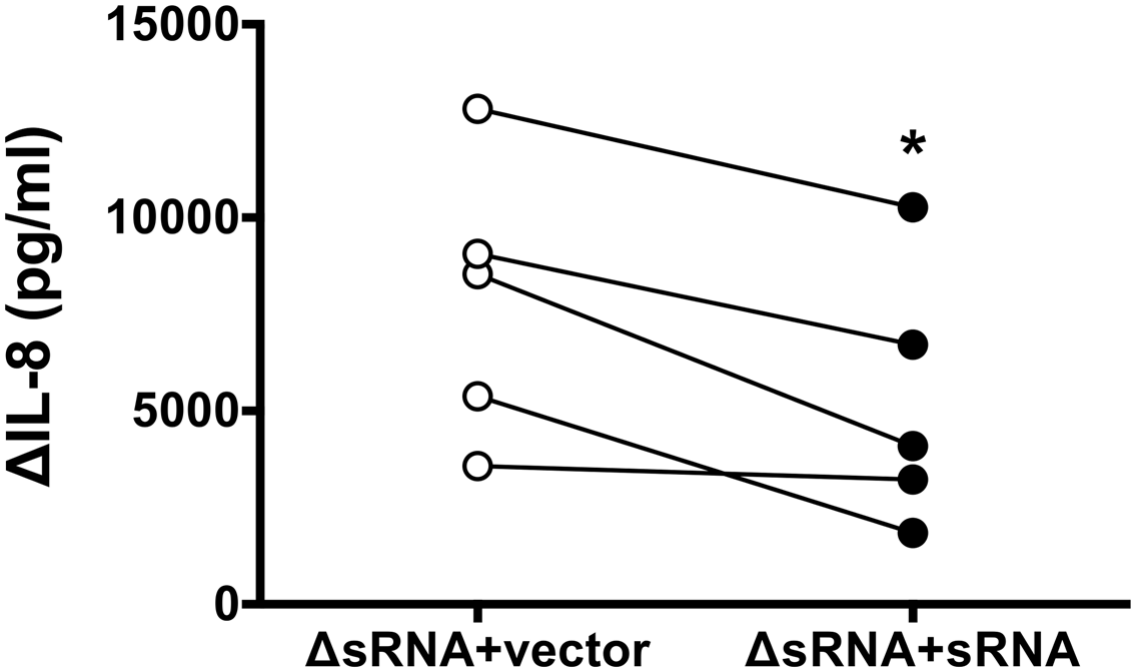
sRNA52320 reduces OMV-stimulated IL-8 secretion by HBE cells. OMV-induced IL-8 secretion was significantly attenuated in HBE cells exposed to ΔsRNA+sRNA OMVs (closed circles) compared to HBE cells exposed to ΔsRNA+vector (open circles). The difference in means of -2645 ± 685 pg/ml was statistically significant (95% CI = -4548 to -743, N = 5, p = 0.018 indicated by an asterisk).

OMV-induced IL-8 secretion was 34% lower in HBE cells exposed to ΔsRNA+sRNA OMVs compared to HBE cells exposed to ΔsRNA+vector OMVs (Fig 6). This is consistent with the conclusion that sRNA52320 reduces OMV-induced IL-8 secretion by human airway epithelial cells. In addition, OMV-induced IL-8 secretion was 40% lower in HBE cells exposed to wt OMVs compared to HBE cells exposed to ΔsRNA OMVs (S4 Fig).

### sRNA52320 reduces OMV-induced secretion of the IL-8 homolog, keratinocyte-derived chemokine (KC), and neutrophil infiltration in mouse lung

**Fig 7:**
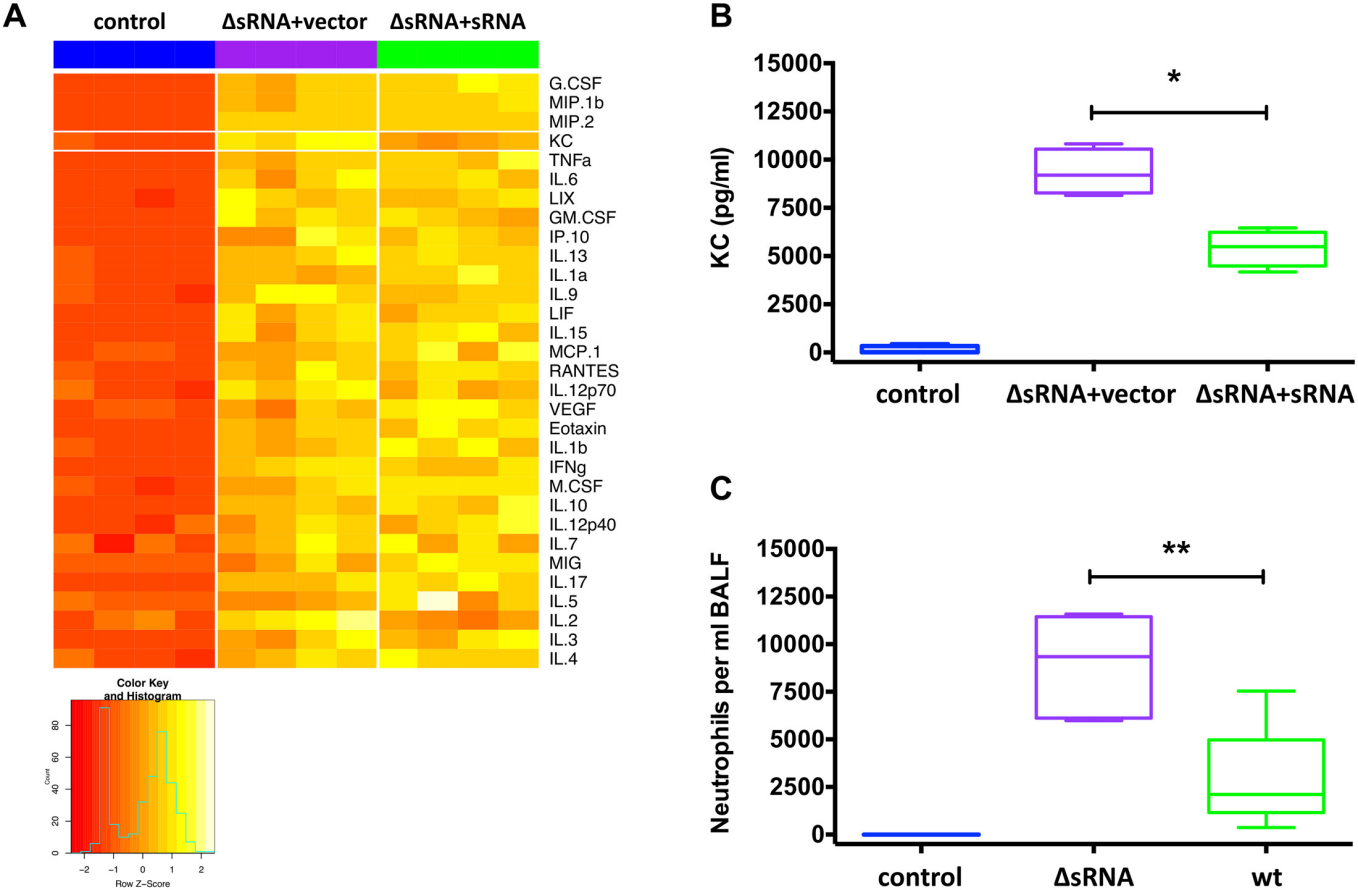
sRNA52320 reduces OMV-induced secretion of the functional IL-8 homolog, KC, and neutrophil infiltration in mouse lung. (A) Heatmap of Z-scores for 31 cytokines in mouse BALF scaled by row (N = 4 mice per group). In each row, relative cytokine abundance ranges from red (low), to orange (medium) to yellow (high). Rows are ordered by mean cytokine abundance with a range of 22,985 pg/ml for G-CSF (top row) to 1.2 pg/ml for IL-4 (bottom row). MIP-1a was excluded from the analysis because it was out of detection range in three of the samples. (B) KC concentration in BALF of mice exposed to vehicle (blue), ΔsRNA+vector OMVs (purple) or ΔsRNA+sRNA OMVs (green). The difference between the means of ΔsRNA+vector OMVs and ΔsRNA+sRNA OMVs of -3933 ± 756 pg/ml was statistically significant (95% CI = -5783 to -2083, N = 4, p = 0.011 indicated by an asterisk). (C) Number of neutrophils per ml BALF of mice exposed to vehicle (blue), ΔsRNA OMVs (purple) or wt OMVs (green). The difference between the means of ΔsRNA OMVs and wt OMVs of -6023 ± 1708 was statistically significant (95% CI = -9962 to -2085, N = 5, p = 0.008 indicated by **).

To determine if sRNA52320 suppresses cytokine secretion *in vivo*, mice were exposed to OMVs isolated from ΔsRNA+vector or ΔsRNA+sRNA *P*. *aeruginosa*. Cytokines were measured in bronchoalveolar lavage fluid (BALF) recovered after a 6 h exposure to OMVs. Vehicle-treated mice served as a negative control. As expected, both types of OMVs induced a cytokine response compared to control mice exposed to vehicle only (Fig 7A and S1 Table). When comparing the effect of ΔsRNA+vector OMVs versus ΔsRNA+sRNA OMVs, KC, a murine functional homolog of IL-8, was the only cytokine that was significantly, and differentially expressed in mouse BALF out of the 31 cytokines measured (S1 Table). KC levels were almost twice as high in BALF from mice exposed to ΔsRNA+vector OMVs compared to mice exposed to ΔsRNA+sRNA OMVs (Fig 7B). This observation is consistent with the conclusion that sRNA52320 selectively reduces OMV-induced KC secretion in mouse lung. Moreover, when mice were exposed to OMVs isolated from ΔsRNA or wt PA14 for 6 h, the number of neutrophils in the BALF of mice exposed to ΔsRNA OMVs was 3-fold higher compared to mice exposed to wt OMVs (Fig 7C), suggesting that OMVs lacking sRNA52320 induced a more robust neutrophil response.

## Discussion

**Fig 8:**
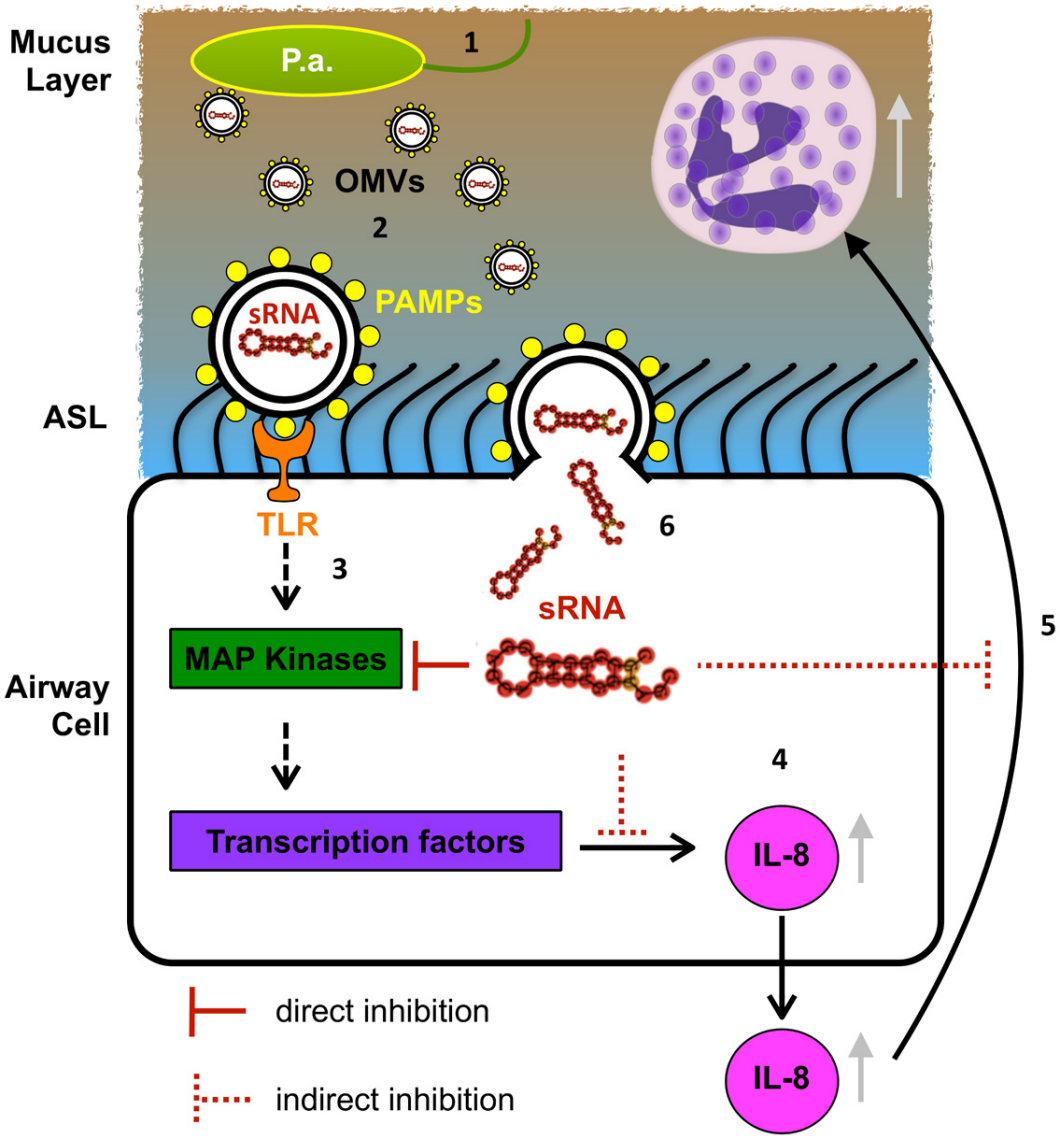
Model of OMV sRNA mechanism of action. (1) *P*. *aeruginosa* (P.a.) resides in the airway mucus layer and produces OMVs. (2) OMVs traverse the mucus layer and reach the airway epithelial cells. (3) Pathogen-associated molecular patterns (PAMPs) on the outside of OMVs induce the host innate immune response by activating the Toll-like receptor and MAP-kinase (TLR/MAPK) signaling pathway. (4) Activation of transcription factors leads to up-regulation of IL-8 mRNA and IL-8 protein secretion. (5) IL-8 is a potent chemoattractant for neutrophils, which infiltrate the lungs and phagocytose *P*. *aeruginosa*. (6) OMVs also fuse with and deliver sRNA52320 into cells, which targets the mRNA of MAP-kinases upstream of IL-8, leading to reduced host IL-8 secretion and neutrophil recruitment. sRNA52320-mediated attenuation of the innate immune response to LPS is a novel mechanism of pathogen-host interaction that may facilitate chronic infection by *P*. *aeruginosa*.

We describe the first example of trans-kingdom biological activity of a regulatory sRNA contained in bacterial OMVs. Our studies demonstrate that sRNA52320 in OMVs secreted by *P*. *aeruginosa* is a novel mechanism of pathogen-host communication that regulates the immune response in human airway epithelial cells and in mouse lung (Fig 8).

In this report we demonstrate for the first time that OMVs produced by an extracellular pathogen deliver sRNAs to host cells. However, the relative abundance of sRNAs in host cells following transfer does not exactly mirror their relative abundance in OMVs. This discrepancy may be explained by differences in RNA transfer efficiency or stability following transfer to the host.

Several recent studies have described the presence of sRNAs in OMVs [33–35], or characterized sRNA produced by intracellular bacteria [39–42], but none to date have demonstrated a role of sRNAs in host cell biology. Furuse et al. identified a 22 nt sRNA in *M*. *marinum*, but intracellular levels of the sRNA were too low to repress target mRNA in cultured cells [39]. Moreover, a high-throughput bioinformatics approach predicted possible targets of bacterial sRNAs in the human transcriptome, and *in vitro* transfection of host cells with a selection of these putative sRNAs decreased target mRNA abundance [43]. However, the actual expression levels of the predicted sRNAs or their effect on a biological response, such as cytokine secretion, were not reported.

OMVs isolated from *P*. *aeruginosa* elicit IL-8 secretion by lung and bronchial epithelial cell lines [14] and increase multiple pro-inflammatory cytokines in a murine macrophage cell line [44] as well as in mouse BALF [15]. In this study we showed that sRNA52320, which was transferred from OMVs to host cells, reduced OMV-stimulated IL-8/KC secretion by human airway epithelia cells and mouse lung and attenuated neutrophil infiltration in a murine model of OMV exposure. Thus, the present study provides the first evidence that sRNAs contained in OMVs target host cell mRNA and result in a reduction of OMV-induced IL-8 (KC) secretion and attenuated recruitment of neutrophils, professional immune cells that phagocytose and kill bacteria. We propose that *P*. *aeruginosa* uses sRNAs, such as sRNA52320, to reduce the ability of the innate immune system to clear *P*. *aeruginosa* from the lungs of infected individuals.

Although the sRNA content of *P*. *aeruginosa* OMVs has not been described previously, multiple groups have used RNA-Seq to characterize the intracellular sRNA content of *P*. *aeruginosa* and found that sRNA expression is highly variable among different strains and growth conditions [23,25,45–47]. Ghosal et al. recently found that OMVs produced by *E*. *coli* were enriched in short RNAs (15–40 nt), and that there was a difference in the profiles of intracellular, OMV-associated and OMV-free extracellular RNA [33]. Moreover, they reported that specific cleavage products of functionally important non-coding RNAs, including tRNAs, constituted a significant portion of the OMV-associated RNA. We also observed differences in the relative expression of sRNAs between OMVs and *P*. *aeruginosa*. Among the top ten most abundant sRNAs in OMVs, four tRNA fragments were significantly more abundant in OMVs compared to *P*. *aeruginosa*, suggesting selective packaging, although nothing is known about the mechanism for differential packaging of sRNAs into OMVs.

tRNA-derived RNA fragments are evolutionarily conserved specific cleavage products found in all domains of life [29,31]. Similar to eukaryotic microRNAs, tRNA fragments have been shown to silence mRNA targets in mammalian cells [29,32]. tRNA fragments are also selectively packaged into human exosomes and may regulate targets in recipient cells [48]. Here we show that sRNA52320, a tRNA-derived fragment that is predicted to target kinases in the LPS-stimulated MAPK signaling pathway, reduces the LPS-mediated induction of IL-8 mRNA and protein secretion in primary human airway epithelial cells and KC production in mouse lung.

Given that the classic TLR-mediated innate immune response to LPS involves the up-regulation of multiple pro-inflammatory cytokines, it is surprising that only IL-8/KC is repressed by sRNA52320. A possible explanation for this observation could be that sRNA52320 predominantly targets mRNA in the TLR2/4-induced innate immune response pathway, while cytokine secretion mediated by other receptors and pathways remains unaffected. For example, knockout of TLR4 and TLR2 blocks the OMV-induced secretion of KC/CXCL-1 more than any of the other cytokines [15]. Likewise, inhibition of TLR2 and TLR4 decreases *Mycobacterium bovis*-induced ERK1/2 activation and subsequent IL-8 secretion in human epithelial cells [49]. Hence, the inhibition of IL-8 and KC secretion by sRNA52320 that we observed in this study is consistent with the hypothesis that sRNA52320 primarily attenuates TLR4 signalling.

In contrast to many other cytokines, KC is secreted in the earliest stages of infection, up to 12 h post-exposure [15], making it one of the key players in the early innate immune response. It has been demonstrated that an effective immune response requires early, KC/IL-8 mediated recruitment of neutrophils to respond to an acute bacterial infection [17]. *P*. *aeruginosa-*mediated attenuation of IL-8 secretion by sRNAs and the resulting reduction in neutrophil infiltration may tip the balance from bacterial clearance towards persistent colonization in individuals with compromised immunity or barrier function. Further investigation beyond the scope of this study is needed to determine if deletion of sRNA52320 does in fact improve bacterial clearance and reduce mortality or chronic infection rates.

The OMV-mediated delivery of sRNAs to host cells might be common to all gram-negative bacteria. It is tempting to speculate that other clinically relevant gram-negative bacteria like *Salmonella*, *E*. *coli*, *Yersinia pestis*, *Klebsiella*, *Shigella*, *Moraxella*, *Helicobacter*, *Acinetobacter*, *Campylobacter*, *Legionella*, *Neisseria* and *Hemophilus* might also use OMV-sRNA based mechanisms to their advantage in the course of infection.

Looking beyond the host-pathogen interaction, OMV sRNAs may also be a mechanism by which gram-negative bacteria compete with other microbes that inhabit the same ecological niche. For example, we identified several stretches of 12–16 nucleotides of sRNA52320 that are antisense to functional genes in other soil bacteria including *Nitrosomonas*, *Nitrobacter*, *Rhizobium*, *Clostridium*, *Methylobacterium*, and *Variovorax paradoxus*. sRNA52320 is also predicted to target genes that affect metabolism, enzymes, transporters and transcription factors in *Streptococcus*, *Rothia*, *Prevotella*, and *Burkholderia*, which often co-colonize susceptible hosts [50]. For example, a target of sRNA52320 in multiple other species of bacteria is the TonB-dependent siderophore receptor, which is important for iron uptake, an essential element for bacterial growth and survival [51].

Future work is needed to elucidate fully the role of OMV sRNAs in microbe-host as well as microbe-microbe interactions and to potentially identify new druggable targets for control of bacterial infections. Once these mechanisms are better understood, the targeted design of sRNA antagonists or the inhibition of OMV production or fusion with host cells might open up new avenues of treatment and the prevention of infections in the face of increasing antibiotic resistance.

## Materials and Methods

### Ethics statement

This study was conducted in strict accordance with the recommendations in the Guide for the Care and Use of Laboratory Animals of the National Institutes of Health. The Dartmouth Institutional Animal Care and Use Committee approved all work with mice (protocol #hoga.da.1). Euthanasia was performed in accordance with the 2013 AVMA Guidelines for the Euthanasia of Animals. Mice were anesthetized with isoflurane during instillation of OMVs into the lung. 6 h after OMV exposure, mice were euthanized with a combination of anesthesia until respiration ceased, followed by cervical dislocation to confirm death.

### *P*. *aeruginosa* cultures and RNA isolation

*P*. *aeruginosa* (strain PA14) was grown in lysogeny broth (LB) as described [52]. For RNA isolation from whole bacteria, 1 ml of an overnight culture was pelleted by centrifugation at 3,300 g for 3 min and washed twice with phosphate-buffered saline (PBS, Thermo Fisher Scientific Inc., Waltham, MA, USA). *P*. *aeruginosa* RNA was isolated with the miRNeasy kit (Qiagen), which retains the small RNA fraction.

### Outer membrane vesicle preparation and RNA isolation

OMVs were isolated as described in [14]. Briefly, for OMV RNA isolation, 35 ml of a PA14 overnight culture was centrifuged for 1 h at 2800 g and 4°C to pellet the bacteria. The OMV-containing supernatant was filtered twice through a 0.45 μm PVDF membrane filter (Millipore, Billerica, MA, USA) followed by ultracentrifugation for 3 h at 200,000 g and 4°C to pellet OMVs. The OMV pellet was washed with OMV buffer (20 mM HEPES, 500 mM NaCl, pH 7.4) and re-pelleted by centrifugation at 200,000 g for 2 h at 4°C. The supernatant was removed and the OMV pellet lysed with Qiazol reagent. OMV RNA was isolated with the miRNeasy kit (Qiagen), which retains the small RNA fraction.

For exposure of mice to OMVs, 50–100 ml of a PA14 overnight culture were pre-processed as described above. OMVs in filtered supernatants were concentrated with 30K Amicon Ultra Centrifugal Filter Units (Millipore, Billerica, MA, USA). Concentrated OMVs were pelleted by ultracentrifugation for 2 h at 46,000 g and 4°C, washed with OMV buffer and re-pelleted. OMV pellets were re-suspended in 60% OptiPrep Density Gradient Medium (Sigma) in OMV buffer and layered with 0.8 ml 40% Optiprep, 0.8 ml 35% Optiprep, 1.6 ml 30% Optiprep and 0.8 ml 20% Optiprep. Samples were centrifuged for 16 h at 100,000 g and 4°C. 500 μl fractions were removed from the top of the gradient, with OMVs residing in fractions 2 and 3, corresponding to 25% Optiprep, as previously shown [14].

### RNA-Seq analysis of PA14 sRNAs

Matched samples of RNA isolated from whole bacteria and the corresponding OMVs were sequenced in three individual preparations. RNA-samples were digested with DNase (DNA-free, Thermo Fisher Scientific) and RNA quality was assessed with a Bioanalyzer (Agilent Technologies, Santa Clara, CA, USA). For each sample, 1 μg DNase-treated total RNA was used for preparation of cDNA libraries with the TruSeq Small RNA Library Preparation Kit (Illumina, San Diego, CA, USA). Libraries were sequenced as 50 bp single-end reads on an Illumina Genome Analyzer.

Reads were trimmed and aligned to the PA14 reference genome (NC_008463.1) using CLC Genomics Workbench (CLC-Bio/Qiagen). The RNA-Seq analysis was run with the following modifications from the standard parameters: a) use of 50 additional bases up- and downstream of annotated genes to capture sRNAs that align to intergenic regions, b) maximum number of mismatches = zero to eliminate unspecific alignment of yeast sequences from the LB medium and c) maximum number of hits for a read = 4. Pileups of uniquely mapped reads as well as frequency tables for each unique sequence (generated with the CLC Small RNA Analysis tool) were exported for normalization and further analysis with the R software environment for statistical computing and graphics [53]. The data discussed in this publication have been deposited in NCBI's Gene Expression Omnibus [54] and are accessible through GEO Series accession number GSE71598 (http://www.ncbi.nlm.nih.gov/geo/query/acc.cgi?acc=GSE71598).

### Bioinformatic sRNA target predictions

RNA secondary structure predictions were obtained for the ten most abundant OMV sRNAs using the RNAfold WebServer [55]. The complementary sequences of the ten most abundant OMV sRNAs were aligned with NCBI human reference sequences using the algorithm BLASTN 2.2.31 [36] to identify perfect matches with human mRNAs. In addition, a miRanda microRNA target prediction scan with a pairing score cutoff of 140 [37] was run for the three most promising candidates for follow-up analysis.

### RNase protection assay

Intact OMVs or OMV RNA (90 ng) were incubated with 10 pg/μl RNase A (Thermo Fisher Scientific) for 1 h at 37°C. Control OMVs were incubated for 1 h at 37°C in the absence of RNase A. To remove RNase, OMVs were washed 3 times with PBS on 30K Amicon Ultra Centrifugal Filter Units (Millipore). OMVs were pelleted by ultracentrifugation at 120,000 g for 70 min. RNA was isolated with the miRNeasy kit (Qiagen) and separated on a 2% agarose gel. RNA was visualized by staining with SYBR Safe (Thermo Fisher Scientific).

### Detection of sRNA52320 by RT-PCR

The presence or absence of sRNA52320 in whole bacteria, OMVs and transfected HBE cells was detected by RT-PCR using the miRCURY LNA Universal RT microRNA PCR system (Exiqon, Woburn, MA, USA). cDNA was synthesized with the Universal cDNA synthesis kit II (Exiqon) according to manufacturer’s instructions. PCR amplification of sRNA52320 was performed using the ExiLENT SYBR Green master mix and custom primers design to specifically target sRNA52320 (Exiqon).

### Cell culture

Human bronchial epithelial (HBE) cells from 6 donors were obtained from Dr. Scott Randell (University of North Carolina, Chapel Hill, NC, USA) and cultured as described previously [56]. Briefly, cells were grown in BronchiaLife basal medium (Lifeline Cell Technology, Frederick, MD, USA) supplemented with the BronchiaLife B/T LifeFactors Kit (Lifeline) as well as 10,000 U/ml Penicillin and 10,000 μg/ml Streptomycin. Culture plates were coated with PureCol Bovine Collagen Solution (Advanced Bio Matrix, Carlsbad, CA, USA). For OMV exposures, HBE cells were polarized on PureCol-coated permeable supports (#3801 Corning Inc., Corning, NY, USA) at an air-liquid interface for 3–4 weeks. During polarization, HBE cells were supplemented on the basolateral side with Air Liquid Interface (ALI) medium [57].

### OMV sRNA transfer to host cells

Polarized HBE cells from two donors were exposed to OMVs containing 1.5 μg of RNA or 20% Optiprep (vehicle ctrl). After incubating for 1 h at 37°C and 5% CO_2_, cells were vigorously washed 5x with PBS and RNA was isolated from HBE cells with the miRNeasy kit (Qiagen). For each sample, 1 μg total RNA was used for preparation of cDNA libraries with the TruSeq Small RNA Library Preparation Kit (Illumina, San Diego, CA, USA). Libraries were sequenced as 50 bp single-end reads on an Illumina HiSeq2500.

Reads were trimmed and aligned to the PA14 reference genome (NC_008463.1) using CLC Genomics Workbench (CLC-Bio/Qiagen). The RNA-Seq analysis was run with the following modifications from the standard parameters: a) Also map to inter-genic regions, b) Mismatch/Insertion/Deletion cost = 3, c) Length/Similarity fraction = 1.0 and d) Maximum number of hits for a read = 1. Pileups of uniquely mapped reads as well as frequency tables for each unique sequence were exported for normalization to library size and further analysis. The data discussed in this publication have been deposited in NCBI's Gene Expression Omnibus [54] and are accessible through GEO Series accession number GSE80421 (http://www.ncbi.nlm.nih.gov/geo/query/acc.cgi?acc=GSE80421).

### Transfection of HBE cells with sRNA52320 and LPS exposure

HBE cells were seeded on PureCol-coated 6-well plates (Corning Inc.) at 300,000 cells per well. Two days after seeding (at 60–70% confluence), cells were switched to antibiotic-free medium and transfected with 10 nM sRNA52320 (Invitrogen custom siRNA, Thermo Fisher Scientific) or 10 nM AllStars Negative Control siRNA (siNC) using HiPerFect transfection reagent (both from Qiagen). Two days after transfection, cells were exposed to 10 μg/ml *P*. *aeruginosa* lipopolysaccharides (LPS, Sigma L8643) for 5 h to induce the release of pro-inflammatory cytokines.

### Proteomic analysis

HBE cells from 3 donors that had been transfected with sRNA52320 or siNC and exposed to LPS were subjected to proteomic analysis. Cells were trypsinized, washed twice with PBS and counted with a cell counter (Bio-Rad Laboratories, Hercules, CA, USA) to ensure that matched samples from each donor had the same number of cells. Each HBE cell pellet was lysed in 8.5 M urea/50 mM Tris pH 8.2 buffer by sonication. Protein content of each lysate was determined by BCA assay (Thermo Fisher Scientific). Proteins were reduced by addition of 5 mM dithiothreitol (DTT) at 50°C for 20 minutes, followed by cooling to room temperature and alkylation with 12.5 mM iodoacetamide for 1 hour in the dark. This reaction was quenched by further addition of 5 mM DTT for 15 minutes at room temperature. The protein samples were diluted 1:5 (vol:vol) in 20 mM Tris, pH 8.2/50 mM NaCl and digested with sequencing grade trypsin (Promega) overnight (1:75, w:w). Protein digests were desalted over C_18_ reverse-phase extraction cartridges (Grace-Vydac), and amounts equivalent to 100 micrograms of peptides were dried in separate tubes by vacuum centrifugation. The dried peptide aliquots were labeled by reductive demethylation as described [58] and fractionated by basic pH reverse-phase fractionation into 12 fractions as described [59]. Each fraction was analyzed by UPLC-MS/MS using a Proxeon LC-1000 UPLC system fitted with an in-house fabricated microcapillary column (100 micron ID x 40 centimeter long) packed with reverse-phase material (Maisch GMBH; Reprosil-Pur C_18_-AQ, 120 Å, 3 micron beads) directly into an Orbitrap Fusion tribrid mass spectrometer (Thermo Fisher Scientific) [60]. The Fusion was operated in data-dependent mode (Orbitrap MS1: R = 120 K, AGC = 2.5e5, max ion injection = 20 ms, scan range = 350–1500 m/z; Orbitrap MS2: R = 15 K, AGC = 5e4, max ion injection = 50 ms, minimum signal = 5e5, charge states = 2–4, normalized HCD energy = 29%, top speed mode cycle time = 2 s). The resultant MS2 scans were data searched using Comet [61], filtered to a 1% peptide false discovery rate (FDR) using the target-decoy strategy [62] and either assigned to unique protein sequences or discarded. Quantification was performed using a modified version of the MassCroQ algorithm [63].

Mean fold changes comparing sRNA52320 with siNC were calculated for each protein that was detected in all 3 replicate samples and p-values were obtained using a one sample t-test. Proteins were ranked by p-value and network analysis of the top 320 proteins was performed with QIAGEN’s Ingenuity Pathway Analysis (IPA, QIAGEN Redwood City, www.qiagen.com/ingenuity). Selection criteria for top canonical pathways were p < 0.05 and absolute activation z-score > 2.

### qPCR for IL-8

For the quantification of HBE IL-8 mRNA levels, 2 μg of total RNA were converted to cDNA using the RETROscript Kit (Thermo Fisher Scientific). 50 ng of template cDNA were used in a TaqMan gene expression assay for IL-8 (#Hs00174103_m1, Thermo Fisher Scientific). GUSB (#Hs99999908_m1) served as endogenous control.

### Construction and culture of *P*. *aeruginosa* sRNA52320 deletion mutant and re-complemented strains

The *P*. *aeruginosa* in-frame sRNA52320 deletion mutant was generated using a construct created via the previously described *Saccharomyces cerevisiae* recombination technique with pMQ30 allelic replacement vector as described in [64]. The following primers were used:

PA14_52320_sRNA_delete_1:

cgcttctgcgttctgatttaatctgtatcaggctgaGTCCGGCCGATAACTGCCATCCAG

PA14_52320_sRNA_delete_2:

GTCCGTAGAATGCGCCCACACAGATCGTCGGGCTCATAACCCGAAGGTC

PA14_52320_sRNA_delete_3:

GACCTTCGGGTTATGAGCCCGACGATCTGTGTGGGCGCATTCTACGGAC

PA14_52320_sRNA_delete_4:

gcggataacaatttcacacaggaaacagctatgGAAGACCGCCGGGTTTTTCAGGAGTTG

In primer sequences, upper case letters indicate *P*. *aeruginosa*-specific sequence. Lower case letters indicate homology with the cloning vector DNA.

For re-complementation of the deletion mutant sRNA52320 was cloned into the arabinose-inducible expression vector pMQ70 [64] using EcoRI and SmaI restriction sites. The cloning was performed by GenScript (GenScript USA Inc., Piscataway, NJ, USA). *P*. *aeruginosa* was transformed with the sRNA52320 expression vector via electroporation, as described previously [65]. *P*. *aeruginosa* transformed with the arabinose-inducible pMQ70 vector and its derivatives were grown in LB with 100 mM arabinose and 300 μg/ml carbenicillin (both from Sigma-Aldrich).

### Characterization of OMVs from engineered *P*. *aeruginosa* strains

LPS content of ΔsRNA+vector OMVs and ΔsRNA+sRNA OMVs was determined using the Pierce LAL Chromogenic Endotoxin Quantitation Kit and total OMV protein content was measured with the Pierce BCA Protein Assay Kit (both Thermo Fisher Scientific) according to manufacturer’s instructions.

### Exposure of polarized HBE cells to OMVs

HBE cells were seeded on PureCol-coated permeable supports (Corning #3801) at 250,000–500,000 cells/filter and polarized at a liquid-air interface for at least 3 weeks. Equal amounts of ΔsRNA+vector OMVs and ΔsRNA+sRNA OMVs (7 μg total protein) were applied to the apical side. After a 6 h exposure, basolateral medium was collected for cytokine measurements.

### Mouse exposure to OMVs

8–9 weeks old male C57BL/6J mice (The Jackson Laboratory, Bar Harbor, ME, USA) were inoculated by oropharyngeal aspiration [66] with OMVs (7 μg total protein) or vehicle following brief anesthesia with isoflurane. OMV protein concentrations were adjusted to keep the total inoculation volume the same for all mice in a given experiment (either 1x or 2x 50 μl). 6 h after exposure, mice were euthanized using a combination of anesthesia until respiration ceased, followed by cervical dislocation to confirm death. In preparation for broncho-alveolar lavage trachea were surgically exposed and catheter tubing (BD #427411, Becton, Dickinson and Company, Franklin Lakes, NJ, USA) fit to a 23 gauge needle (BD #305145) was inserted into the trachea and stabilized with a single suture (#100–5000, Henry Schein Inc., Melville, NY, USA). BAL fluid (BALF) was collected by flushing 1 ml of sterile PBS into the lungs and recovered from the lungs with a syringe (BD #309659). This process was repeated once.

### Cytokine measurements

Cytokine secretion from HBE cells was measured with a PromoKine Human IL-8 ELISA Development Kit (PromoCell GmbH, Heidelberg, Germany). Cytokines in mouse BALF were detected with the Millipore mouse cytokine 32-plex kit (EMD Millipore Corporation, Billerica, MA).

### Quantification of neutrophils in mouse BALF

The total number of cells in each BALF sample was counted and samples were adjusted to 750,000 cells per ml. 200,000 cells per sample were added to the cytospin apparatus and centrifuged onto glass slides at 700 rpm for 5 minutes at room temperature. Slides were dried and stained with the Differential Quik Stain Kit (Polysciences, Warrington, PA) according to the included protocol. Neutrophils were enumerated under 100x magnification using an Olympus IX-73 microscope. Original neutrophil numbers per ml of BALF were calculated by accounting for the dilution factors used to adjust cells to 750,000/ml.

### Statistical analysis

Statistical analysis was performed using the R software environment for statistical computing and graphics [53] and GraphPad Prism 6 for Mac OS X software version 6.0h. Differences between experimental and control groups were evaluated using a two-tailed unpaired Student’s t-test where appropriate and are reported as differences in means ± SEM. For experiments using primary HBE cells paired t-tests or mixed effect linear models with donor as a random effect were used to account for donor-to-donor variability. Statistical significance of mouse cytokine data was determined with ANOVA followed by Tukey’s post-hoc test.

### Accession numbers

sRNA52320 (NCBI locus tag PA14_RS21305), IL-8 (P10145), KC/CXCL-1 (P12850), MAP2K2 (P36507), MAP2K3 (P46734), MAP2K4 (P45985), MAP3K7 (O43318) and PIK3R2 (O00459).

## Supporting Information

S1 Fig Differential sRNA52320 read alignment of RNA sequences obtained from OMVs and *P*. *aeruginosa*. (A) Read alignment for OMV RNA to the *PA14_52320* locus, which codes for tRNA-Met. Uniquely mapped sequences are green, while sequences that map to multiple loci in the *P*. *aeruginosa* genome are shown in yellow. For OMV RNA uniquely mapped sequences are predominant in the first half of the tRNA. sRNA52320 comprises the first 24 nucleotides of tRNA-Met and has the following sequence: 5’-CGCGGGAUGGAGCAGUCUGGUAGC-3’. (B) Read alignment for *P*. *aeruginosa* whole cell RNA to the *PA14_52320* locus. In contradistinction to OMV RNA samples, RNA isolated from *P*. *aeruginosa* has a high number of unique reads (green) mapping to both the 5’- and 3’ ends of tRNA-Met.
(TIFF)

S2 Fig Extracellular sRNA52320 does not permeate or affect IL-8 secretion by HBE cells. HBE cells were incubated with 10 nM sRNA52320 in the presence or absence of HiPerfect transfection reagent (lanes 2 and 3). HBE cells transfected with siNC (indicated as a -) served as a negative control (lane 1). (A) In the presence of transfection reagent, sRNA52320 could be detected inside of HBE cells (lane 2), whereas in the absence of transfection reagent extracellular sRNA52320 was not detectable in lysed host cells (lane 3). (B) sRNA52320 (filled black circles) significantly reduced LPS-stimulated IL-8 secretion compared to control (open circles). By contrast, in the absence of transfection reagent extracellular sRNA52320 had no effect on IL-8 secretion (grey circles). Statistical significance was determined with a mixed effect linear model with donor as a random effect. Asterisk indicates p = 0.039.
(TIFF)

S3 Fig Characterization of OMVs isolated from the sRNA52320 deletion mutant and the re-complemented strain. (A) PCR for sRNA52320 confirms the absence of sRNA52320 in the ΔsRNA+vector knockout strain (left lane) as well as the presence of sRNA52320 in the re-complemented ΔsRNA+sRNA strain (right lane). (B) sRNA52320 levels were similar in wt OMVs (grey squares) and ΔsRNA+sRNA OMVs (filled circles). The difference in the mean Cts was not statistically significant (N = 6 means of 3 technical replicates each). (C) There was no significant difference in LPS content of ΔsRNA+vector OMVs (open circles) and ΔsRNA+sRNA OMVs (filled circles). (D) The protein content of ΔsRNA+vector OMVs (open circles) was similar to ΔsRNA+sRNA OMVs (filled circles).
(TIFF)

S4 Fig wt OMVs stimulate less HBE IL-8 secretion than ΔsRNA OMVs. OMV-induced IL-8 secretion was significantly attenuated in HBE cells exposed to wt OMVs (closed circles) compared to HBE cells exposed to ΔsRNA OMVs (open circles). The difference in means of -159 ± 49 pg/ml was statistically significant (95% CI = -285 to -33, N = 6, p = 0.02 indicated by an asterisk).
(TIFF)

S1 Table KC is uniquely regulated by sRNA52320. p-values for comparisons of 31 cytokines in BALF obtained from control mice and mice exposed to ΔsRNA+vector OMVs or ΔsRNA+sRNA OMVs were obtained from one-way ANOVA with a Tukey HSD post-hoc test followed by Bonferroni correction for multiple comparisons. Comparisons with a corrected p-value < 0.05 were considered significant and are highlighted in bold. The murine IL-8 homolog KC was the only cytokine with a significant difference in abundance between mice exposed to ΔsRNA+vector OMVs versus ΔsRNA+sRNA OMVs.
(DOCX)
