# Supplementary figures and images for: A Novel Mechanism of Host-Pathogen Interaction through sRNA in Bacterial Outer Membrane Vesicles
Source: PLoS Pathog. 2016 Jun 13;12(6):e1005672. doi: 10.1371/journal.ppat.1005672 (PMC4905634; doi:10.1371/journal.ppat.1005672)

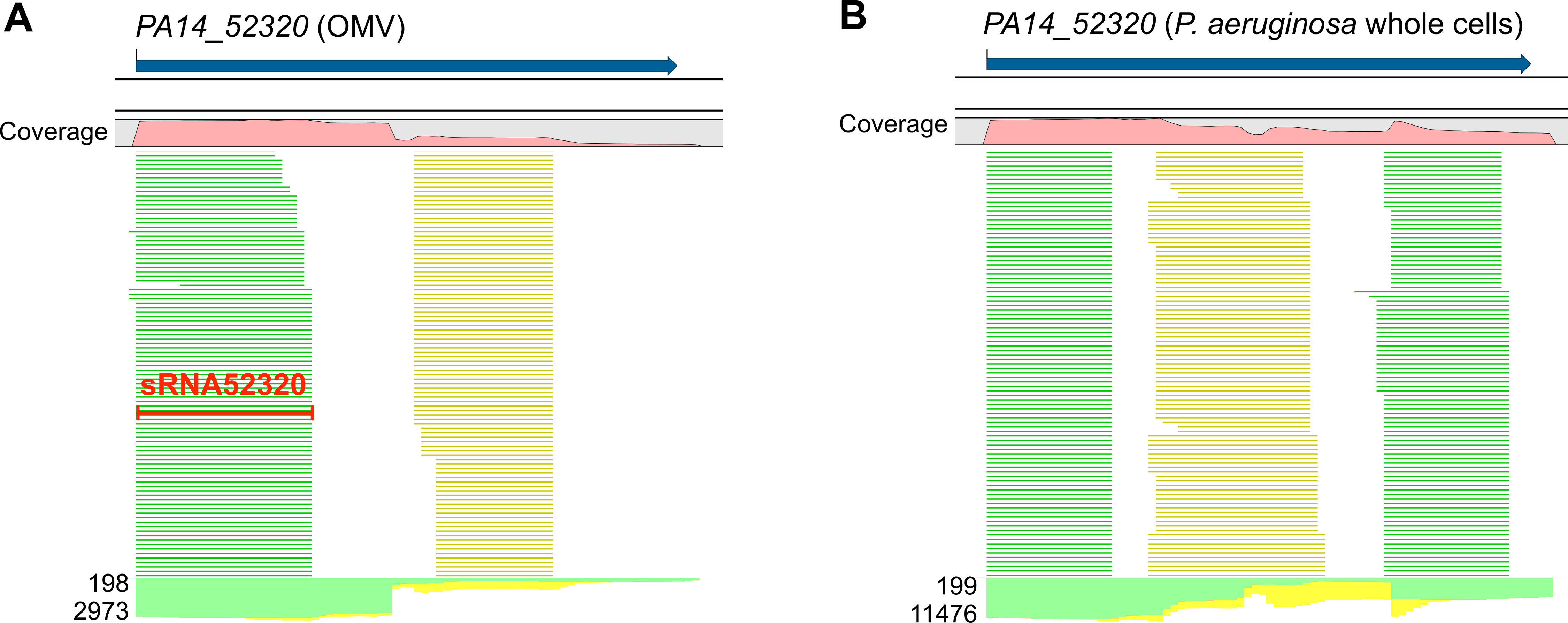

Supplement: S1 Fig — (A) Read alignment for OMV RNA to the PA14_52320 locus, which codes for tRNA-Met. Uniquely mapped sequences are green, while sequences that map to multiple loci in the P. aeruginosa genome are shown in yellow. For OMV RNA uniquely mapped sequences are predominant in the first half of the tRNA. sRNA52320 comprises the first 24 nucleotides of tRNA-Met and has the following sequence: 5’-CGCGGGAUGGAGCAGUCUGGUAGC-3’. (B) Read alignment for P. aeruginosa whole cell RNA to the PA14_52320 locus. In contradistinction to OMV RNA samples, RNA isolated from P. aeruginosa has a high number of unique reads (green) mapping to both the 5’- and 3’ ends of tRNA-Met. (TIFF) [file ppat.1005672.s001.tiff]

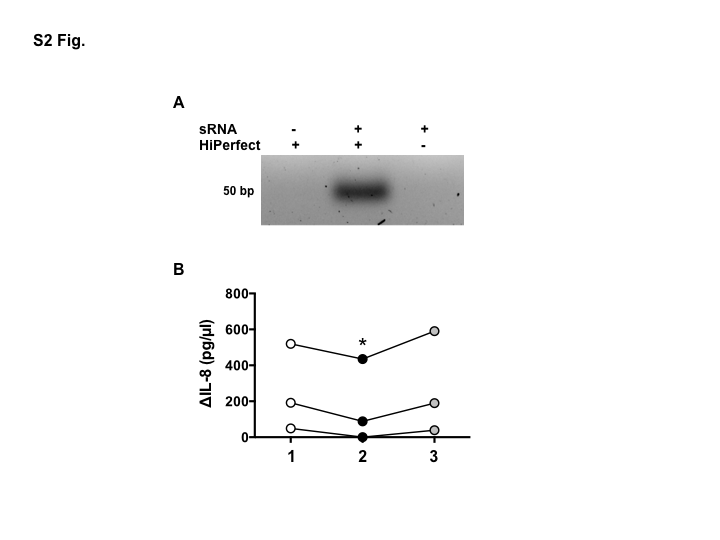

Supplement: S2 Fig — HBE cells were incubated with 10 nM sRNA52320 in the presence or absence of HiPerfect transfection reagent (lanes 2 and 3). HBE cells transfected with siNC (indicated as a -) served as a negative control (lane 1). (A) In the presence of transfection reagent, sRNA52320 could be detected inside of HBE cells (lane 2), whereas in the absence of transfection reagent extracellular sRNA52320 was not detectable in lysed host cells (lane 3). (B) sRNA52320 (filled black circles) significantly reduced LPS-stimulated IL-8 secretion compared to control (open circles). By contrast, in the absence of transfection reagent extracellular sRNA52320 had no effect on IL-8 secretion (grey circles). Statistical significance was determined with a mixed effect linear model with donor as a random effect. Asterisk indicates p = 0.039. (TIFF) [file ppat.1005672.s002.tiff]

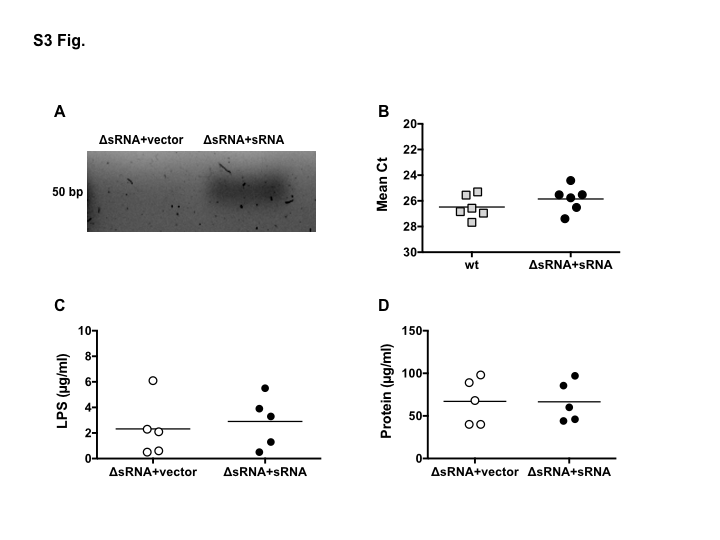

Supplement: S3 Fig — (A) PCR for sRNA52320 confirms the absence of sRNA52320 in the ΔsRNA+vector knockout strain (left lane) as well as the presence of sRNA52320 in the re-complemented ΔsRNA+sRNA strain (right lane). (B) sRNA52320 levels were similar in wt OMVs (grey squares) and ΔsRNA+sRNA OMVs (filled circles). The difference in the mean Cts was not statistically significant (N = 6 means of 3 technical replicates each). (C) There was no significant difference in LPS content of ΔsRNA+vector OMVs (open circles) and ΔsRNA+sRNA OMVs (filled circles). (D) The protein content of ΔsRNA+vector OMVs (open circles) was similar to ΔsRNA+sRNA OMVs (filled circles). (TIFF) [file ppat.1005672.s003.tiff]

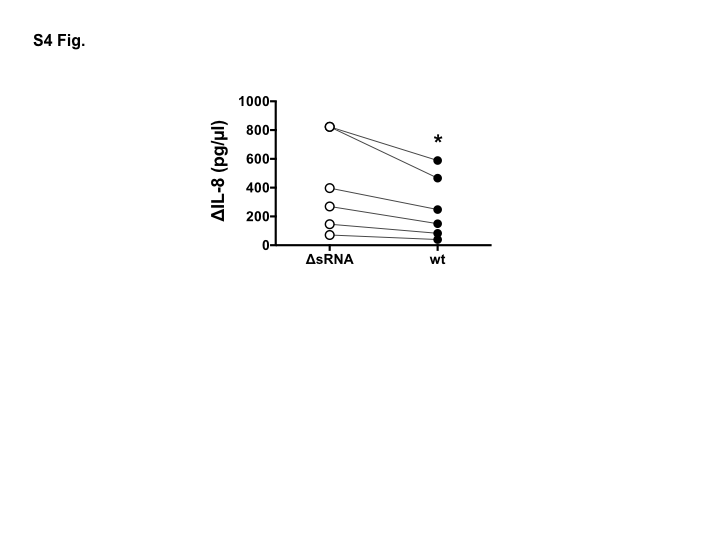

Supplement: S4 Fig — OMV-induced IL-8 secretion was significantly attenuated in HBE cells exposed to wt OMVs (closed circles) compared to HBE cells exposed to ΔsRNA OMVs (open circles). The difference in means of -159 ± 49 pg/ml was statistically significant (95% CI = -285 to -33, N = 6, p = 0.02 indicated by an asterisk). (TIFF) [file ppat.1005672.s004.tiff]
