# Supplementary material for: A Novel Mechanism of Host-Pathogen Interaction through sRNA in Bacterial Outer Membrane Vesicles
Source: PLoS Pathog. 2016 Jun 13;12(6):e1005672. doi: 10.1371/journal.ppat.1005672 (PMC4905634; doi:10.1371/journal.ppat.1005672)
Supplement: S1 Table — p-values for comparisons of 31 cytokines in BALF obtained from control mice and mice exposed to ΔsRNA+vector OMVs or ΔsRNA+sRNA OMVs were obtained from one-way ANOVA with a Tukey HSD post-hoc test followed by Bonferroni correction for multiple comparisons. Comparisons with a corrected p-value < 0.05 were considered significant and are highlighted in bold. The murine IL-8 homolog KC was the only cytokine with a significant difference in abundance between mice exposed to ΔsRNA+vector OMVs versus ΔsRNA+sRNA OMVs. (DOCX) [file ppat.1005672.s005.docx]

**S1 Table. KC is uniquely regulated by sRNA52320.**

| Cytokine | ΔsRNA+vector vs. ctrl | ΔsRNA+sRNA vs. ctrl | ΔsRNA+sRNA vs. ΔsRNA+vector |
| --- | --- | --- | --- |
| Eotaxin | **0.00061** | **0.00016** | 1.00000 |
| G.CSF | **0.00162** | **0.00028** | 1.00000 |
| GM.CSF | **0.00139** | **0.00556** | 1.00000 |
| IFNg | **0.00004** | **0.00006** | 1.00000 |
| IL.1a | **0.00479** | **0.00021** | 0.66223 |
| IL.1b | **0.00127** | **0.00012** | 1.00000 |
| IL.2 | **0.01065** | 1.00000 | 0.20124 |
| IL.3 | 0.10754 | **0.03845** | 1.00000 |
| IL.4 | **0.02658** | **0.00774** | 1.00000 |
| IL.5 | 1.00000 | 0.39399 | 1.00000 |
| IL.6 | **0.00482** | **0.00438** | 1.00000 |
| IL.7 | 0.17930 | 0.20040 | 1.00000 |
| IL.9 | **0.00144** | **0.00545** | 1.00000 |
| IL.10 | **0.00540** | **0.00090** | 1.00000 |
| IL.12p40 | 0.13664 | **0.03886** | 1.00000 |
| IL.12p70 | **0.00392** | **0.02721** | 1.00000 |
| IL.13 | **0.00220** | **0.00117** | 1.00000 |
| IL.15 | **0.00486** | **0.00208** | 1.00000 |
| IL.17 | **0.00018** | **0.00002** | 1.00000 |
| IP.10 | 0.13970 | 0.14321 | 1.00000 |
| KC | **0.00001** | **0.00114** | **0.01132** |
| LIF | **0.00215** | **0.00242** | 1.00000 |
| LIX | **0.00018** | **0.00037** | 1.00000 |
| MCP.1 | 0.16316 | **0.01049** | 1.00000 |
| M.CSF | **0.00160** | **0.00019** | 1.00000 |
| MIG | 0.15069 | **0.00295** | 0.82595 |
| MIP.1b | **0.00011** | **0.00002** | 1.00000 |
| MIP.2 | **0.00000** | **0.00000** | 1.00000 |
| RANTES | **0.00572** | **0.00165** | 1.00000 |
| TNFa | **0.00478** | **0.00093** | 1.00000 |
| VEGF | 0.08020 | **0.00143** | 0.54019 |

p-values for comparisons of 31 cytokines in BALF obtained from control mice and mice exposed to ΔsRNA+vector OMVs or re-complemented ΔsRNA+sRNA OMVs were obtained from one-way ANOVA with a Tukey HSD post-hoc test followed by Bonferroni correction for multiple comparisons. Comparisons with a corrected p-value < 0.05 were considered significant and are highlighted in bold. The murine IL-8 homolog KC was the only cytokine with a significant difference in abundance between mice exposed to ΔsRNA+sRNA OMVs and ΔsRNA+vector OMVs.
